# Cost-Effectiveness Analysis of Ultrasound Screening for Thyroid Cancer in Asymptomatic Adults

**DOI:** 10.3389/fpubh.2021.729684

**Published:** 2021-09-22

**Authors:** Nan Yang, Han Yang, Jeff Jianfei Guo, Ming Hu, Sheyu Li

**Affiliations:** ^1^West China School of Pharmacy, Sichuan University, Chengdu, China; ^2^James L. Winkle College of Pharmacy, University of Cincinnati Academic Health Center, Cincinnati, OH, United States; ^3^Department of Endocrinology and Metabolism, West China Hospital, Sichuan University, Chengdu, China; ^4^Chinese Evidence-Based Medicine Center, Cochrane China Center and MAGIC China Center, West China Hospital, Sichuan University, Chengdu, China

**Keywords:** ultrasound screening, thyroid cancer, Markov model, cost-effectiveness, asymptomatic adults

## Abstract

**Objectives:** This study evaluated the long-term cost-effectiveness of ultrasound screening for thyroid cancer compared with non-screening in asymptomatic adults.

**Methods:** Applying a Markov decision-tree model with effectiveness and cost data from literature, we compared the long-term cost-effectiveness of the two strategies: ultrasound screening and non-screening for thyroid cancer. A one-way sensitivity analysis and a probabilistic sensitivity analysis were performed to verify the stability of model results.

**Results:** The cumulative cost of screening for thyroid cancer was $18,819.24, with 18.74 quality-adjusted life years (QALYs), whereas the cumulative cost of non-screening was $15,864.28, with 18.71 QALYs. The incremental cost-effectiveness ratio of $106,947.50/QALY greatly exceeded the threshold of $50,000. The result of the one-way sensitivity analysis showed that the utility values of benign nodules and utility of health after thyroid cancer surgery would affect the results.

**Conclusions:** Ultrasound screening for thyroid cancer has no obvious advantage in terms of cost-effectiveness compared with non-screening. The optimized thyroid screening strategy for a specific population is essential.

## Introduction

Thyroid cancer is one of the most common malignancies, which accounts for 1 to 1.5% of all malignant tumors in the United States (1). With the rapid development of the ultrasound technique in the primary care, the incidence of thyroid cancer exploded in the past few decades including in Korea and many other countries (2–4). However, the increase of incidence and prevalence does not company with increasing disease-specific fatality (5–7). The International Agency for Research on Cancer under the World Health Organization and other relevant expert organizations agree that the rising incidence of thyroid cancer in many countries, especially in high-income countries, is largely caused by over-diagnosis and numerous false-positive cases (8), with ultrasound screening being the most widely used method of diagnosing thyroid cancer. Relevant studies and recommendations were issued to reduce and prevent this phenomenon. In 2017, the United States Preventive Services Task Force on thyroid cancer screening online, did not endorse thyroid cancer ultrasound screening for asymptomatic adults because of the lack of evidence (9). In addition, Korean studies demonstrated that thyroid cancer screening did not reduce the thyroid cancer-related fatality (10). Although active surveillance of thyroid cancer is call in low-risk people with incidental thyroid neoplasm, most individuals go with surgical removal given inadequate evidence confirmed their lifelong safety without surgery. Over-diagnosis put people at the unnecessary risk of thyroid surgery such as hoarseness and primary hypoparathyroidism without a balanced benefit, leading to unnecessary labeling of lifelong diagnosis and unnecessary treatments (11).

Further, thyroid cancer ultrasound screening overdrew the finance of healthcare system with accumulated diagnosed cases. There are some studies reported the economic burden and value of thyroid cancer ultrasound screening (12, 13). A study in 2013 showed ultrasound screening for thyroid cancer was cost-effective in selected obese patients (14). However, it is not clear whether screening for all asymptomatic individuals has the advantage of cost-effectiveness compared with the cost in the long run. This study aimed to evaluate the long-term effectiveness of ultrasound screening for thyroid cancer in asymptomatic adults taking the United States as example through decision-tree Markov models, facilitating the decision making for clinicians and policy makers.

## Materials and Methods

### Model Design

We established a Markov decision-tree model using the decision analysis software (TreeAge Pro 2011; TreeAge Software, Williamstown, MA, USA) and developed two different strategies: thyroid cancer ultrasound screening and non-screening according to the disease progression and treatment prognosis. For the screening strategy, all asymptomatic populations aged ≥20 years (15, 16), underwent neck ultrasound screening which revealed healthy status (no nodules), benign nodules (follow-up with no treatment), or malignant nodules (follow-up and treatment). For the non-screening strategy, individuals underwent routine physical examinations (palpation), which also showed healthy status (no nodules), benign nodules (follow-up with no treatment), or malignant nodules (follow-up and treatment).

There were some assumptions underlying the use of these two strategies. First, considering that all Koreans underwent thyroid cancer screening in 2008, we used the Korean thyroid cancer epidemiological data for 1990 and 2010 to determine the transition probabilities for the screening group and the non-screening group, respectively, assuming that the natural incidence of thyroid cancer had remained unchanged between these years (17–19). Second, we assumed that the cost of routine physical examinations for the non-screening group was lower than that of ultrasound examinations for the screening group. Third, we assumed that individuals in the two groups were initially aged 20 years because thyroid disease in children differs from that in adults (16). Accordingly, we used the average disease incidence because the mobility and mortality for different ages were difficult to obtain, but we considered age-wise differences in natural mortality. Fourth, we did not consider any treatments for postoperative complications.

**Figure 1:**
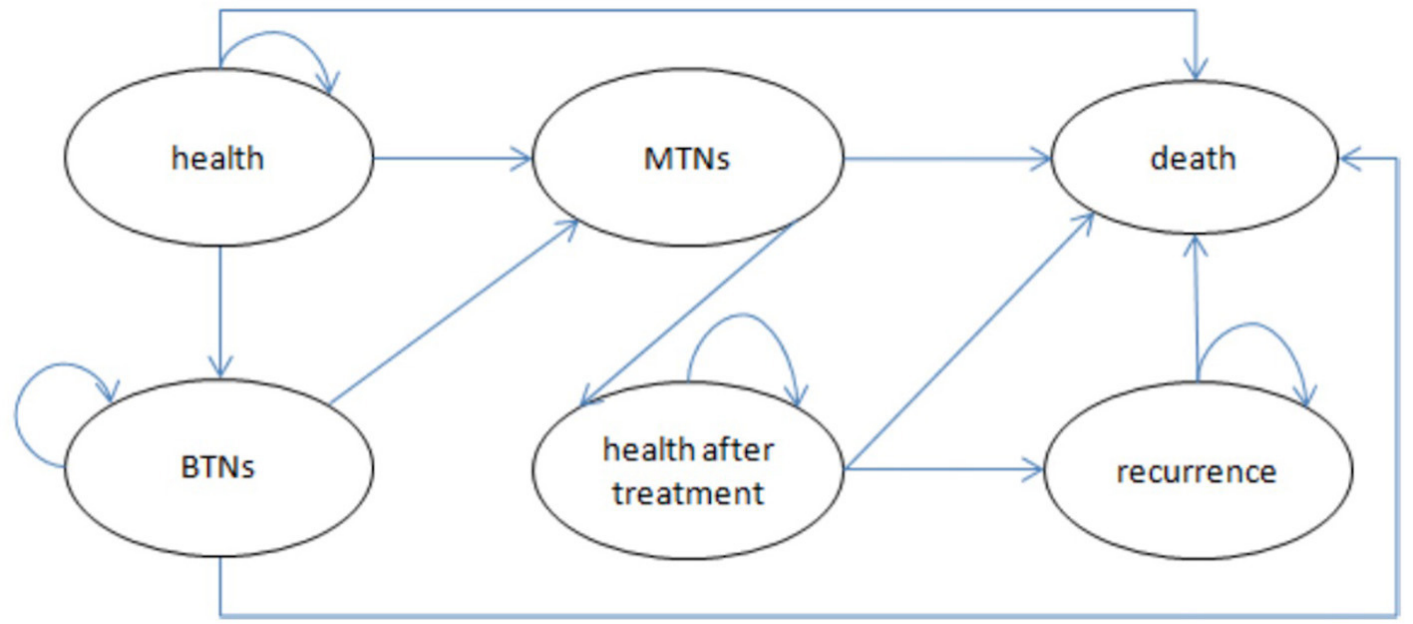
Bubble chart of Markov model. BTNs, benign thyroid nodules (state); MTNs, malignant thyroid nodules (state).

We compared long-term cost-effectiveness of the screening group and non-screening strategies in this study. Because we were unable to obtain the relevant data on thyroid nodules and thyroid cancer in different disease statues, we simply divided thyroid nodules into benign and malignant (thyroid cancer) and established a basic model. The transition probabilities in the model were calculated on the basis of the results of published clinical trials and official data procured from the Korea Statistical Office (20–24). Follow-ups for the screening group and non-screening groups were both lifelong. The quality of life and financial burden of patients were closely related to the recurrence of thyroid cancer. Therefore, we used recurrence as an absorbing state to provide a more accurate description of differences in the effectiveness and costs of these two strategies. The model ran over a 55-year time horizon according to the disease characteristics and life expectancy of Koreans (21); healthy individuals could live as long as their projected life expectancy. Figure 1 presents a simplified disease status model.

### Effectiveness

We included six health statuses were included in the models: recurrence, thyroid cancer postoperative stability, thyroid cancer, benign thyroid nodules, health, and death. The effectiveness was reported in quality-adjusted life years (QALYs), which were calculated by multiplying the utility values for each health state by the duration of health state. Utilities were derived from studies on the quality of life of patients with thyroid cancer, and all future QALYs were discounted 5% annually. Table 1 presents the specific utility values.

**Table 1:** Health utility values and associated model variables.

**Variable**	**Base-case**	**Range**	**Distribution**	**Source**
Thyroid cancer recurrence	0.54	0.49–0.59	Beta (α: 176, β: 150)	Selberherr et al. (25)
Thyroid cancer postoperative stability	0.99	0.74–1.00	Beta (α: 1, β: 0)	Selberherr et al. (25)
Thyroid cancer	0.60	0.54–0.66	Beta (α: 153, β: 102)	The United States Cancer Statistics (26)
Benign thyroid nodules	0.99	0.89–1.00	Beta (α: 100, β: 1)	Selberherr et al. (25)
Perfect health	1.00			
Death	0			

### Costs

In our model, we calculated total costs from the perspective of the whole society, encompassing the costs of examination, surgery, drugs, follow-ups (once a year for life), and productivity losses. Hospitalization costs and labor losses caused by thyroid cancer were calculated on the basis of expert recommendations and guidelines for various countries, according to which 1 to 2 days of hospitalization are required for patients who undergo thyroid cancer surgery, followed by approximately 2 weeks for the recovery, the hospitalization costs, and labor losses of thyroid cancer were estimated (26). All costs, which are listed in Table 2 were expressed in U.S. dollars, using the dollar value in 2017, and an inflation rate equal to the mean of the annual changes in the Consumer Price Index for Medical Care since the year of the reported cost was applied (26). All future costs were discounted 5% annually.

**Table 2:** Cost variables for the modeling.

**Cost component**	**Base-case**	**Range**	**Distribution**	**Source**
c_t	20,174.5786	16,737.47–24,283.81	Gamma (α: 29, β: 706)	(26–29)
c_n	251.46	126.06–311.52	Gamma (α: 7, β: 34)	(26–29)
c_tf	1,264.9032	731.99–2309.31	Gamma (α: 3, β: 492)	(26–29)
c_nf	798.6	399.30–1597.20	Gamma (α: 2, β: 449)	(26–29)
c_recurrence	6,050.22	3,024.78–91.00	Gamma (α: 4, β: 1513)	(26–29)
c_o	50.16	25.08–75.24	Gamma (α: 4, β: 13)	(26–29)

*c_t, thyroid cancer treatment costs; c_recurrence, thyroid cancer recurrence costs; c_tf, thyroid cancer postoperative follow-up costs; c_nf, thyroid benign nodules follow-up costs; c_n, thyroid ultrasound screening costs; c_o, general physical examination costs*.

### Outcomes

We calculated cumulative costs and effectiveness by performing Markov queue simulations, and simulation results of the model were expressed as cost-effectiveness ratio (CER) and incremental cost-effectiveness ratio (ICER). If the cost was lower and the effect was better, then a strategy entailing a smaller CER was the recommended. If the cost was low and the effect was also poor, then the ICER was calculated and compared with the set threshold value. If it exceeded the threshold, then the cost was lower, and if it was below the threshold, then the higher-cost solution was selected. Because the cost of this study was expressed in U.S. dollars, we set the threshold at $50,000/QALY in light of recommendations made in several medical decision analysis studies conducted in the United States (14).

### Transition Probability

The transition probability between disease states in the model was obtained from clinical trials, standardized follow-up trials, and official data of the Korean Bureau of statistics (13). By using the incidence or transition probability of different disease states in 1990 and 2010, respectively, the event probability in 1-year period was calculated by using the formula 1 given below:


(1) $$\begin{array}{r} tp=1-{\left(1-tp_{t}\right)}^{1/t} \end{array}$$

where tp is the transition probability and *t* is the time.

The transition probability included the incidence rate of benign nodules, the incidence rate of malignant nodules, the probability of benign nodule developing into malignant nodules, mortality of malignant nodules, recurrence rate of malignant nodules, and natural mortality rate. This study assumed that the natural incidence rate of thyroid cancer is comparable across ethnicities. The probability of benign nodules developing into malignant nodules was the same in the two groups, and the incidence of malignant nodules is different from the result of screening. So the incidence rate of malignant nodules was different in two groups due to screening. With the increase of the detection rate of thyroid cancer, the mortality also increased. In addition, it is found that the surgical treatment of thyroid cancer is the same, and the postoperative recurrence rate should be the same. Due to the increased number of cancers found, the cancer mortality had also increased.

In addition, the recurrence rates were the same in two groups after the same surgical treatment of thyroid cancer. The specific values and distribution of transition probability are listed in Table 3.

**Table 3:** Probability variables used in the modeling.

**Variable**	**Group**	**Base-case**	**Range**	**Distribution**	**Source**
Health → BTNs	SG	0.19	0.14–0.24	Beta (α: 50, β: 211)	
	NSG	0.68	0.51–0.85	Beta (α: 19, β: 9)	
Health → MTNs	SG	0.000039	0.000029–0.000049	Beta (α: 62, β: 1575948)	Kwong et al. (15)
	NSG	0.000583	0.000437–0.000729	Beta (α: 61, β: 105337)	Kwong et al. (15)
BTNs → MTNs	SG	0.00854478	0.00640859–0.01068098	Beta (α: 61, β: 7070)	Kwong et al. (15)
	NSG	0.00854478	0.00640859–0.01068098	Beta (α: 61, β: 7070)	Kwong et al. (15)
MTNs → death	SG	0.000003	0.00000195–0.00000325	Beta (α: 62, β: 23640522)	Teng
	NSG	0.000005	0.000004–0.000007	Beta (α: 62, β: 11175470)	Teng
Health after treatment → Recurrence	SG	0.001029	0.000772–0.001286	Beta (α: 61, β: 59621)	Wang et al. (22)
	NSG	0.001029	0.000772–0.001286	Beta (α: 61, β: 59621)	Wang et al. (22)

*BTNs, benign thyroid nodules (state); MTNs, malignant thyroid nodules (state); SG, screening group; NSG, non-screening group*.

### Sensitivity Analyses

A one-way sensitivity analysis was performed in relation to the probabilities of all costs, utility values, and transitions to determine the effects of different values of variables and uncertainties on the model results. The range of values for the variables was determined on the basis of those used for sensitivity analyses in previous studies, as shown in Tables 1–3.

A probabilistic sensitivity analysis entailing Monte Carlo simulations was set to simulate 1,000 times. We assumed that the transfer probability and utility value were beta distribution, and the cost was gamma distribution.

For the parameters with available value ranges, the values were assigned according to the upper and lower parameter limits. According to the previous literature and our own research experience, for the parameters whose value range was not available, a basic value is selected ±10% was selected. The specific parameters are shown in Tables 1–3.

## Results

### Results of the Base-Case Analysis

The results of a long-run simulation of the status of thyroid cancer metastasis revealed that the cumulative cost of screening for thyroid cancer was $18,819.24, with 18.74 QALYs, whereas the cumulative cost of non-screening was $15,864.28 with 18.71 QALYs. The ICER of the two groups was $106,947.5/QALY, which greatly exceeded the set threshold value of $50,000/QALY (Table 4). Although both the cost and utility of thyroid cancer screening were higher than those for non-screening, they were unacceptable relative threshold values, and non-screening for thyroid cancer was determined to be a better cost-effective strategy. The difference between the cumulative utility values of the two strategies was evidently nominal, and the costs of screening were much higher, so the cost-effectiveness advantage of the screening group was not significant.

**Table 4:** Summary findings of a cost-effectiveness analysis of thyroid cancer screening data.

**Strategy**	**Total Cost**	**Total QALY**	**Incremental Cost**	**Incremental QALY**	**ICER**
Screening	$18,819.24	18.74	2,954.96	0.03	106,947.5
Non-screening	$15,864.28	18.71			

*QALY, quality-adjusted life years; ICER, incremental cost-effectiveness ratio*.

### Sensitivity Analyses

**Figure 2:**
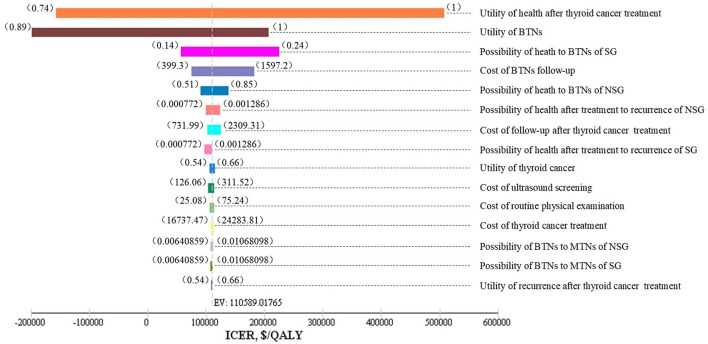
Tornado diagram. SG, screening group; NSG, non-screening group; BTNs, benign thyroid nodules (state); MTNs, malignant thyroid nodules (state); EV, expected value.

As shown in Figure 2, one-way sensitivity analyses were performed for all costs, probabilities, and utility values. The utility values of health after thyroid cancer surgery, and benign nodules; follow-up costs of benign thyroid nodules; incidences of benign nodules; and some other parameters impacted on the model results. The lower utility value of benign nodules and the utility value of health after thyroid cancer surgery could have influenced the result, making a screening strategy more cost-effective.

**Figure 3:**
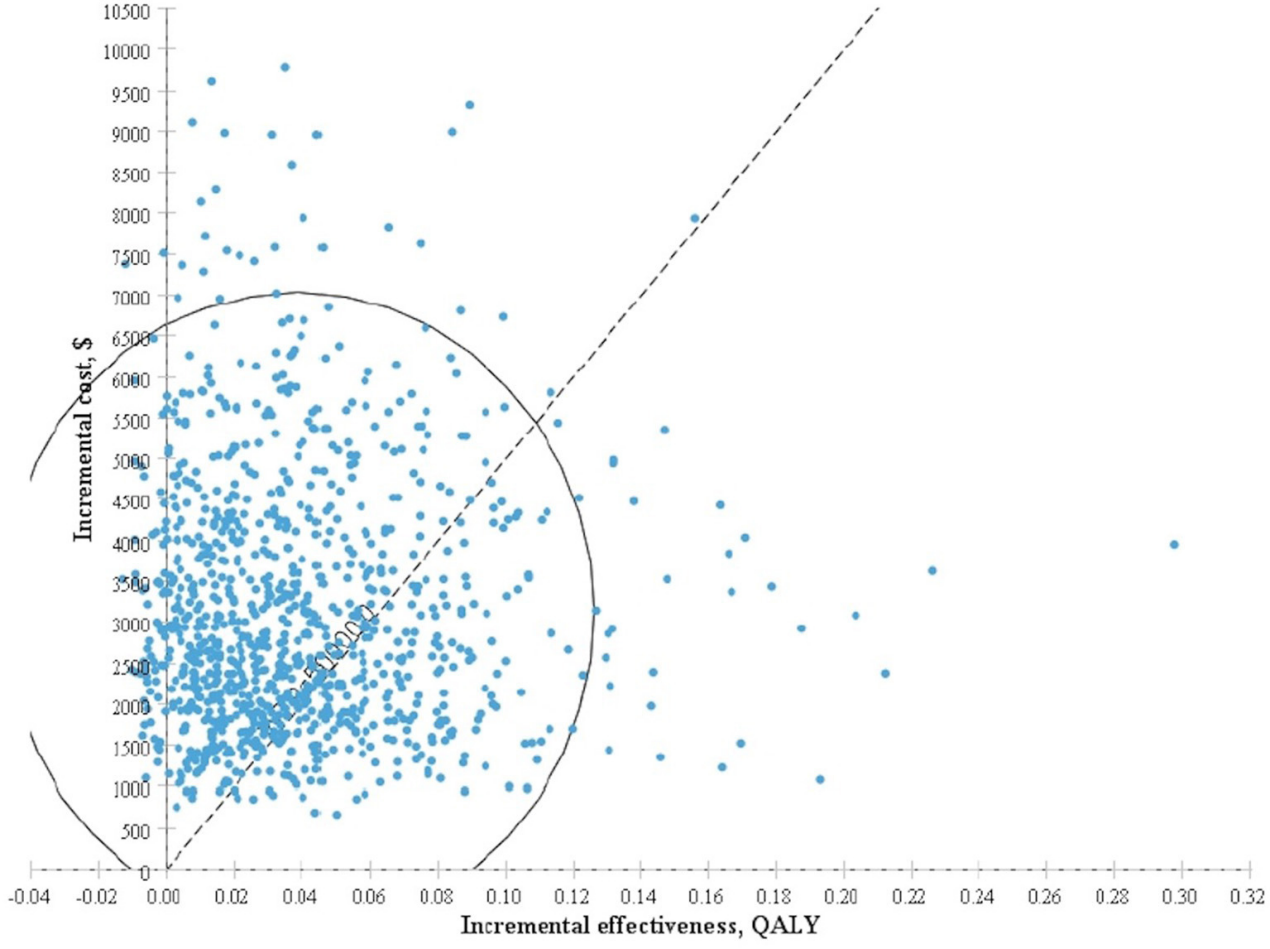
Incremental cost-effectiveness scatter plot for screening group vs. non-screening group based on Monte Carlo simulation.

**Figure 4:**
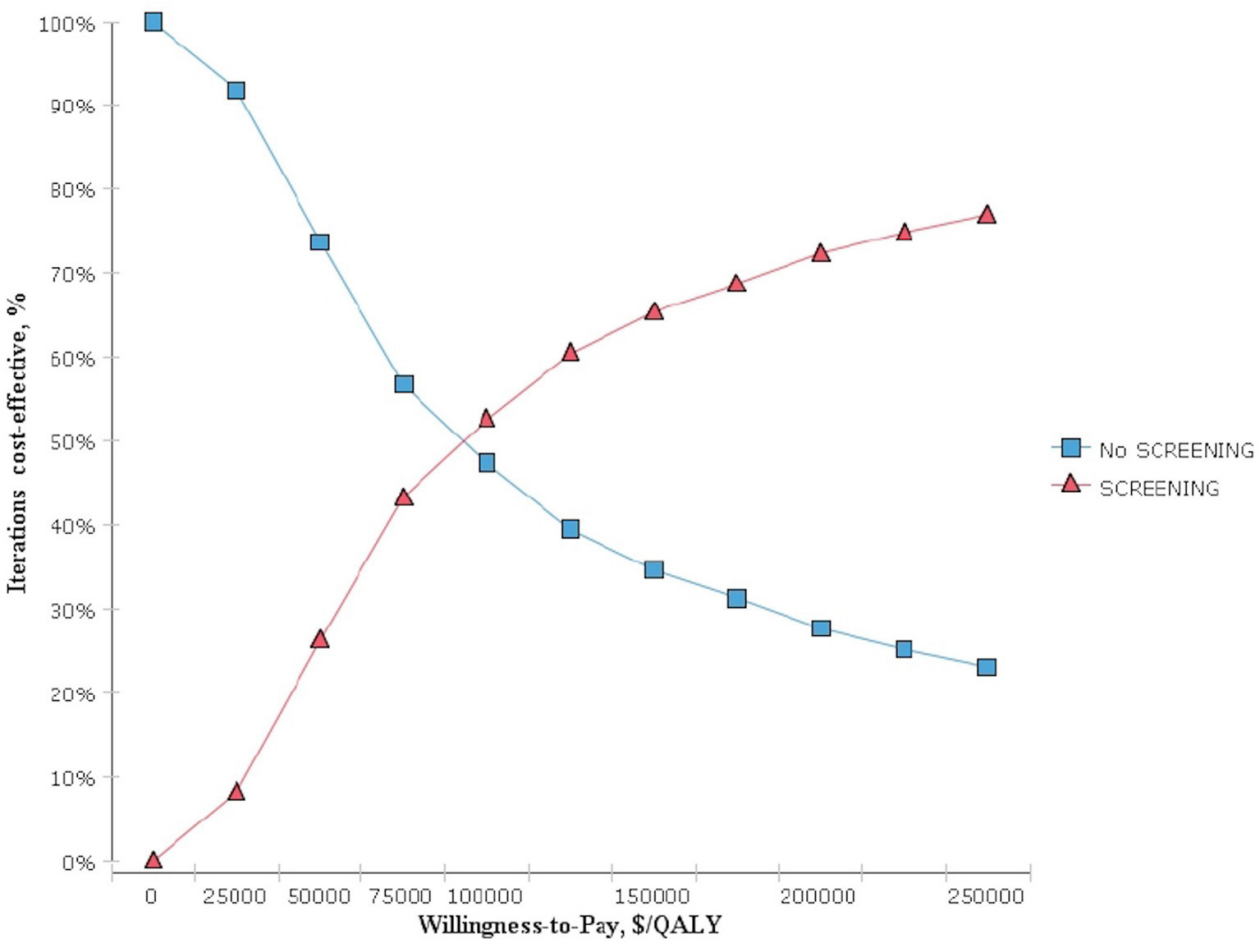
Monte Carlo simulation cost-effectiveness acceptability curve.

The results obtained after performing 1,000 Monte Carlo simulations were consistent with those of the cohort simulation. Figure 3, which depicts a Monte Carlo simulation scatter plot, reveals most of the sites in the 1,000 simulation analyses fell within the first quadrant. In other words, both the cost and effectiveness of the screening group was exceeded those of the non-screening group. About 70% of the sites were located in the willingness to pay, indicating a 70% probability that non-screening for thyroid cancer was more cost-effective compared with screening. The acceptable cost-effectiveness curve is shown in Figure 4. The acceptability of the screening group was higher than that of non-screening group when the willingness to pay was higher than $115,000. The results of the sensitivity analysis showed that a non-screening strategy was more cost-effective at a set threshold value and confirmed the reliability of our results.

## Discussion

This study showed that thyroid screening obtained 18.74 QALYs, and gained 0.03 QALYs more than the non-screening population, with an incremental cost of $2,954.96 incurred for screening. In the setting of this study, ultrasound screening of thyroid cancer is undoubted cost-effectiveness compared with non-screening in asymptomatic adults. Thyroid cancer identified by ultrasound screening in general population may not be that which impairs people's lives. Without such screening, many people may go peacefully with their thyroid nodules in their lifelong time. This quantitative result informed the policy maker to prevent such screening in the aspect of the society.

Although our study are against the thyroid cancer screening the whole asymptomatic population under the model background of this study, thyroid cancer screening may be useful in selected people. Thyroid cancer screening for specific populations, may be cost-effectiveness in selected conditions (14). In addition, ultrasound tests are necessary in people with suspected thyroid nodules or at higher risks of thyroid malignancy. The clinical practice guideline needs to consider both the values and preferences and local baseline characteristics of the population.

According to the results of our sensitivity analysis, follow-up costs associated with benign thyroid nodules impacted on the results of our model. In this model, patients were required to undergo regular lifelong follow-up with very low recurrent rate of thyroid cancer. The follow-up strategy for people with thyroid nodules may need proper exploration and investigation. In addition, postoperative monitoring may also be excessive and may generate unnecessary costs. Research conducted on the annual monitoring costs of papillary thyroid cancer indicates that annual postoperative surveillance of the patients is excessive and may contribute to unnecessary costs. One study evaluated the cost-effectiveness of tapering postoperative surveillance to 3-year intervals after 5 years of annual surveillance in place of perpetual annual follow-ups for patients with low-risk papillary thyroid cancer who demonstrated excellent therapeutic responses (29). The model and sensitivity analyses of our study are consistent with the results of this study, which also provide economic evidence for postoperative monitoring of thyroid cancer.

There are several limitations to be acknowledged. First, because of the lack of studies on the health utility values for patients with benign thyroid nodules, we estimated the value for patients with benign thyroid nodules according to their disease status and life status when inputting model parameters, assigning the same health utility value to these patients and postoperative patients. However, patients with benign nodules may experience negative moods after identifying the nodule due to cancerophobia or other stresses. Although thyroid nodules do not compromise health of an individual, negative emotions, such as anxiety, can lead to diminished health benefits but unmeasurable in the current study. Second, all the costs in this study were derived from the literature, and can be out-of-date when our results are in used. However, our sensitivity analyses confirmed the robustness of the results within acceptable changes of the costs.

## Conclusion

Ultrasound screening for thyroid cancer in an asymptomatic population is not cost-effective, and inappropriate in asymptomatic adults. Policy makers need to be cautious with unregulated thyroid cancer screening in avoidance of the waste of healthcare resource. Further studies are needed to keep the screening and follow-up strategy up to date.

## Data Availability Statement

The original contributions presented in the study are included in the article/Supplementary Material, further inquiries can be directed to the corresponding author/s.

## Author Contributions

NY and HY developed the Markov model and analyzed the data collected from literature and wrote the manuscript. MH made substantial contributions to the conception and design of the study and reviewed and edited the manuscript. SL conceived the study, participated in the coordination and the acquisition of data, and helped to draft the manuscript. JG co-reviewed and co-edited the manuscript. All authors have read and approved the final manuscript.

## Funding

This research was supported by the 111 Project (Grant No. B18035) and Chinese Cardiovascular Association-Access fund (Grant No. 2019-CCA-ACCESS-103). The funders are not involved in the analysis and interpretation of the data or in the decision to publish the results.

## Conflict of Interest

The authors declare that the research was conducted in the absence of any commercial or financial relationships that could be construed as a potential conflict of interest.

## Publisher's Note

All claims expressed in this article are solely those of the authors and do not necessarily represent those of their affiliated organizations, or those of the publisher, the editors and the reviewers. Any product that may be evaluated in this article, or claim that may be made by its manufacturer, is not guaranteed or endorsed by the publisher.

## Abbreviations

QALYs: quality-adjusted life years
CER: cost-effectiveness ratio
ICER: incremental cost-effectiveness ratio.
