# Supplementary material for: Cost-Effectiveness Analysis of Ultrasound Screening for Thyroid Cancer in Asymptomatic Adults
Source: Front Public Health. 2021 Sep 22;9:729684. doi: 10.3389/fpubh.2021.729684 (PMC8494179; doi:10.3389/fpubh.2021.729684)
Supplement: Supplementary file 1 [file Table_1.DOCX]

**Supplementary 1**

**Cost Unit($)**

| **Category** | **Item** | **Base-Case** | **Lower Limit** | **Upper Limit** | **Reference** |
| --- | --- | --- | --- | --- | --- |
| Surgery | Hemithyroidectomy (direct costs) | 5,679.00 | 4,542.91 | 6,814.37 | [2-4] |
|  | Hemithyroidectomy (indirect costs) | 684.00 | 546.48 | 822.10 | [2-4] |
|  | Total thyroidectomy, direct costs | 7,266.00 | 5,811.70 | 8,719.92 | [2-4] |
|  | Total thyroidectomy, indirect costs | 993.00 | 793.58 | 1,192.75 | [2-4] |
| Hospitalization | Hospitalization cost for thyroid operation | 6,263.00 | 5,636.70 | 6,889.30 | [2-4] |
|  | Two-day hospitalization for calcium supplementation | 2,684.88 | 2,416.39 | 2,953.37 | [2-4] |
| Productivity Lost | Cost of 2 weeks of lost productivity (per week) | 1,147.87 | $1,033.08 | 1,262.66 | [1] |
| Medicine | Levothyroxine supplementation after total thyroidectomy (per week) | 5.11 | 4.59 | 5.62 | [2-4] |
|  | Levothyroxine supplementation after thyroid lobectomy (per week) | 4.36 | 3.92 | 4.79 | [2-4] |
|  | Yearly cost of TSH | 485.00 | 244.20 | 970.20 | [2-4] |
| Follow-Up | Yearly cost of surveillance | 1,020 | 511.50 | 2,039.40 | [2-4] |
|  | Yearly cost of follow-up of benign nodules | 799.00 | 399.30 | 1,597.20 | [2-4] |
| Examination | Radioactive iodine ablation (whole-body 131I scan, 131I ablation, one inpatient day) | 451.00 | 226.00 | 902.00 | [2-4] |
|  | FNA biopsy | 159.00 | 79.86 | 172.92 | [2-4] |
|  | Tg test | 97.71 | 97.71 | $97.71 | [2] |
|  | Tg Ab test | 96.76 | 96.72 | $96.76 | [2] |
|  | Routine follow-up | 50.00 | 25.08 | $75.24 | [2-4] |
|  | Neck ultrasound | 92.00 | 46.20 | $138.60 | [2-4] |
| Recurrence | Reoperation for recurrence | 6,050.00 | 3,024.78 | $9,075.00 | [2-4] |

**Reference**

1. The United States Cancer Statistics. https://www.cdc.gov/cancer/thyroid/. [accessed 25 Jun, 2020 ].
2. Lang BH, Wong CKH. A cost-effectiveness comparison between early surgery and non-surgical approach for incidental papillary thyroid microcarcinoma. European Journal of Endocrinology. (2015) 173:367-75.doi: 10.1530/EJE-15-0454
3. Borget I, Bonastre J, Catargi B, Deandreis D, Zerdoud S, Rusu D, et al. Quality of life and cost-effectiveness assessment of radioiodine ablation strategies in patients with thyroid cancer: results from the randomized phase III ESTIMABL trial. Journal of Clinical Oncology. (2015) 33:2885-92.doi: 10.1200/JCO.2015.61.6722.
4. Haser GC, Tuttle RM, Su HK, Alon EE, Bergman D, Bernet V, et al. Active surveillance for papillary thyroid microcarcinama: new challenges and opportunities for the health care system. Endocr Pract. (2016) 22:602-11.doi: 10.4158/EP151065.RA.
